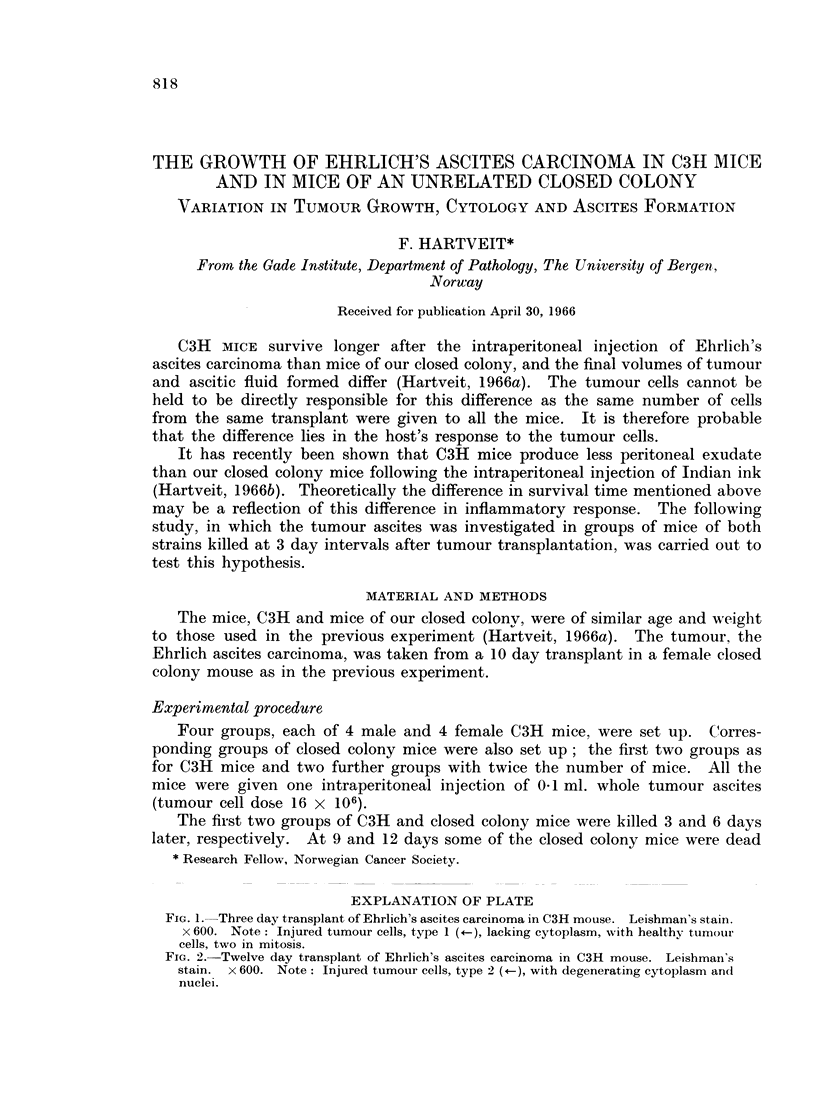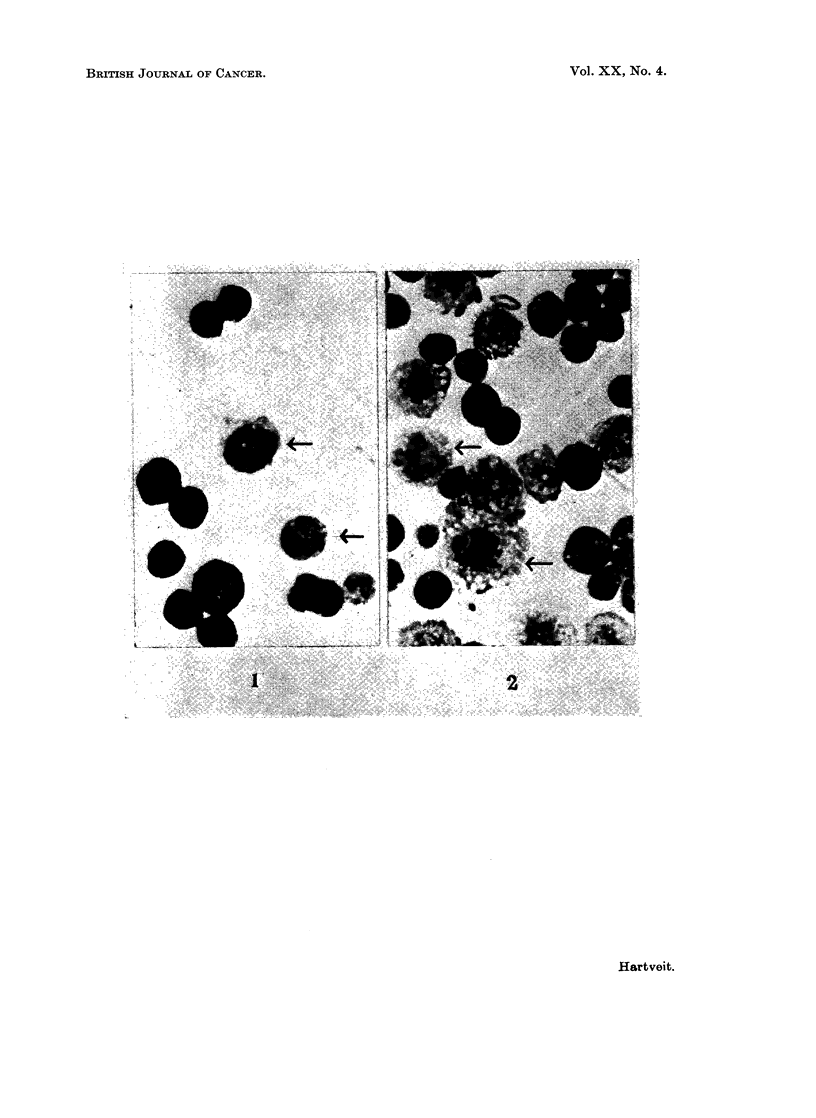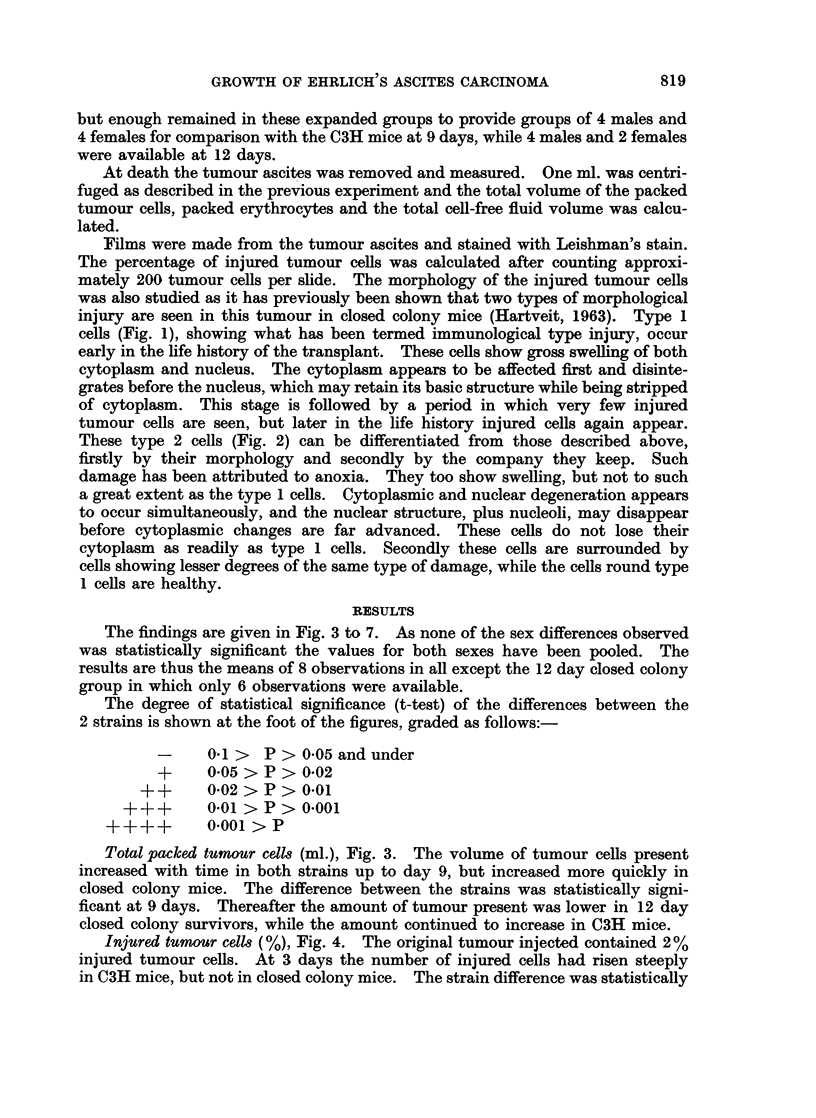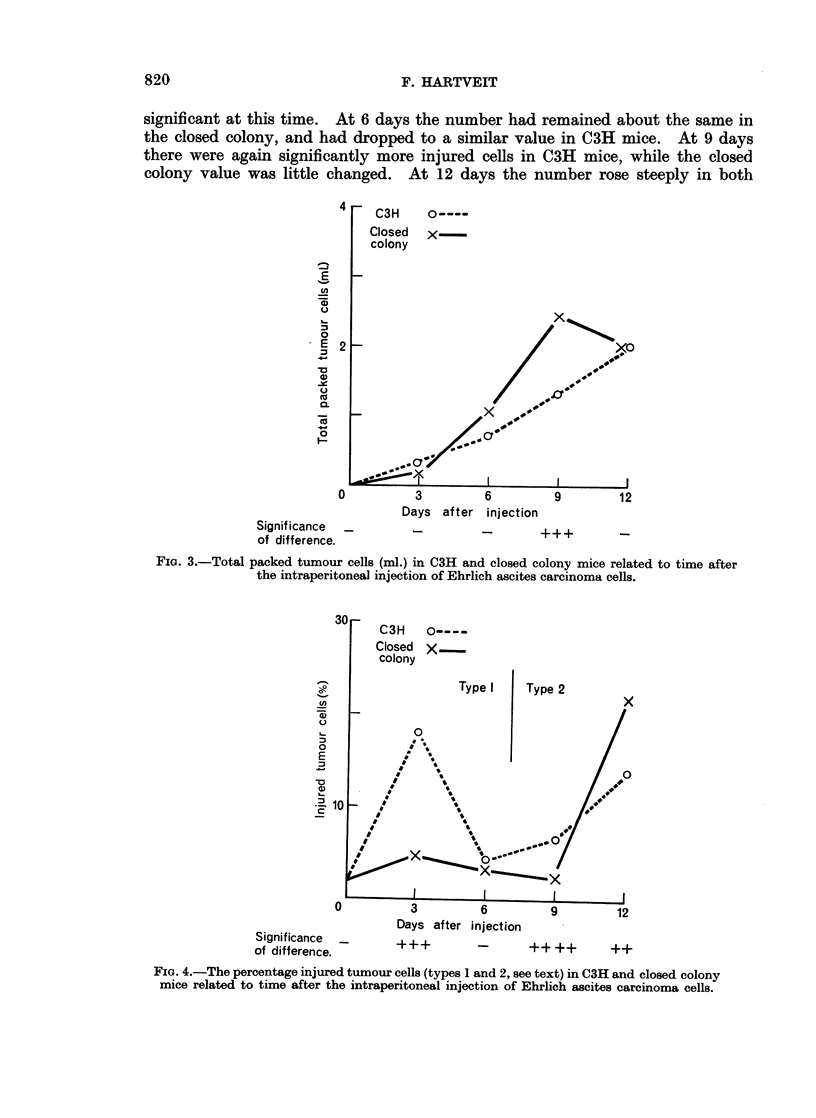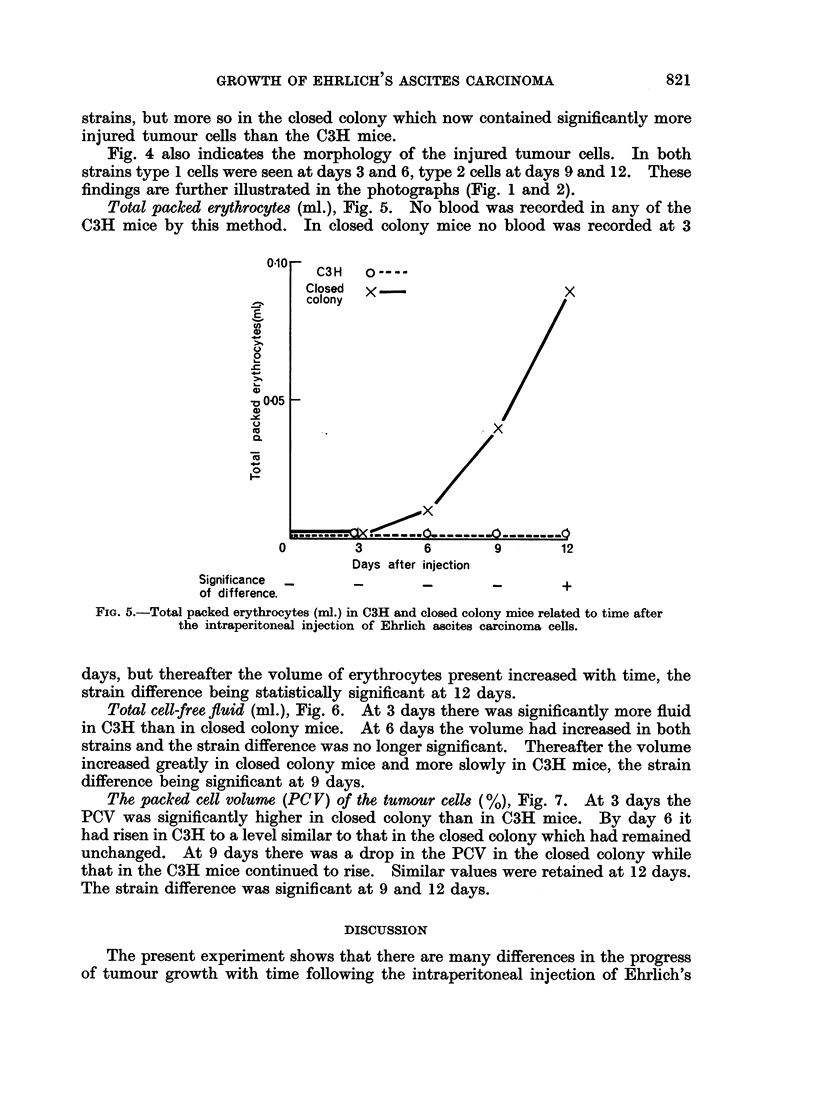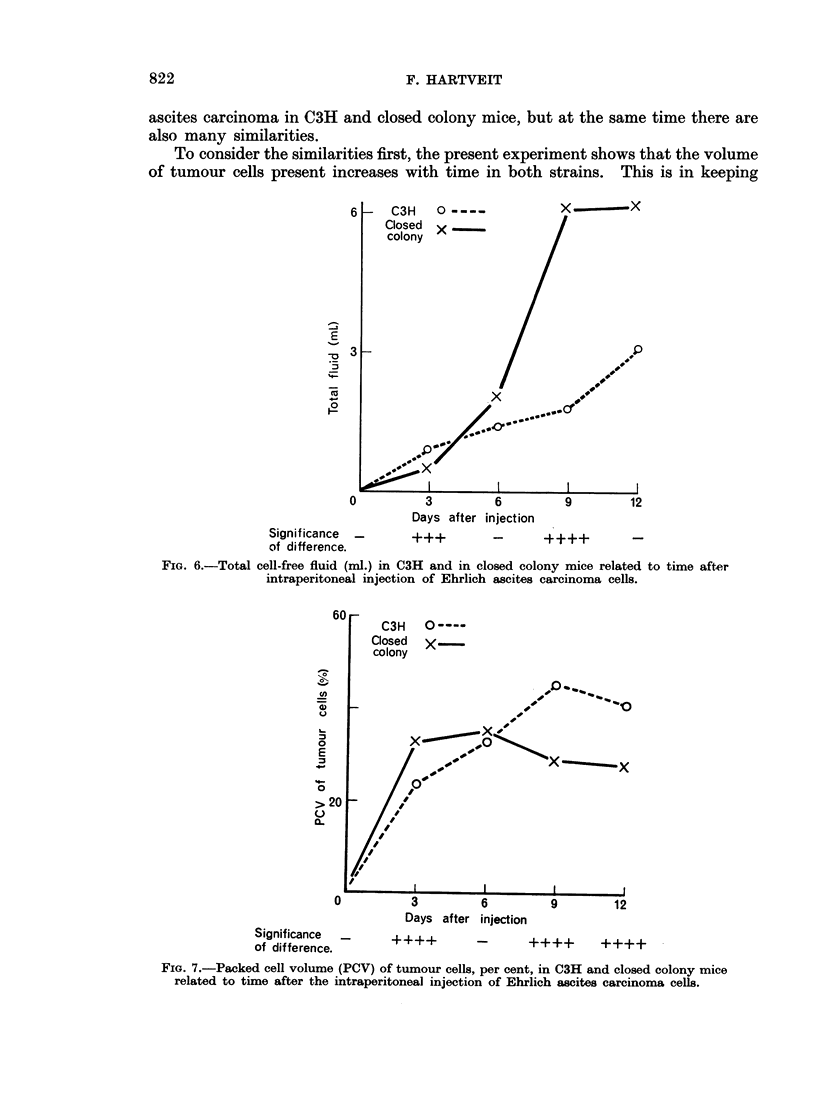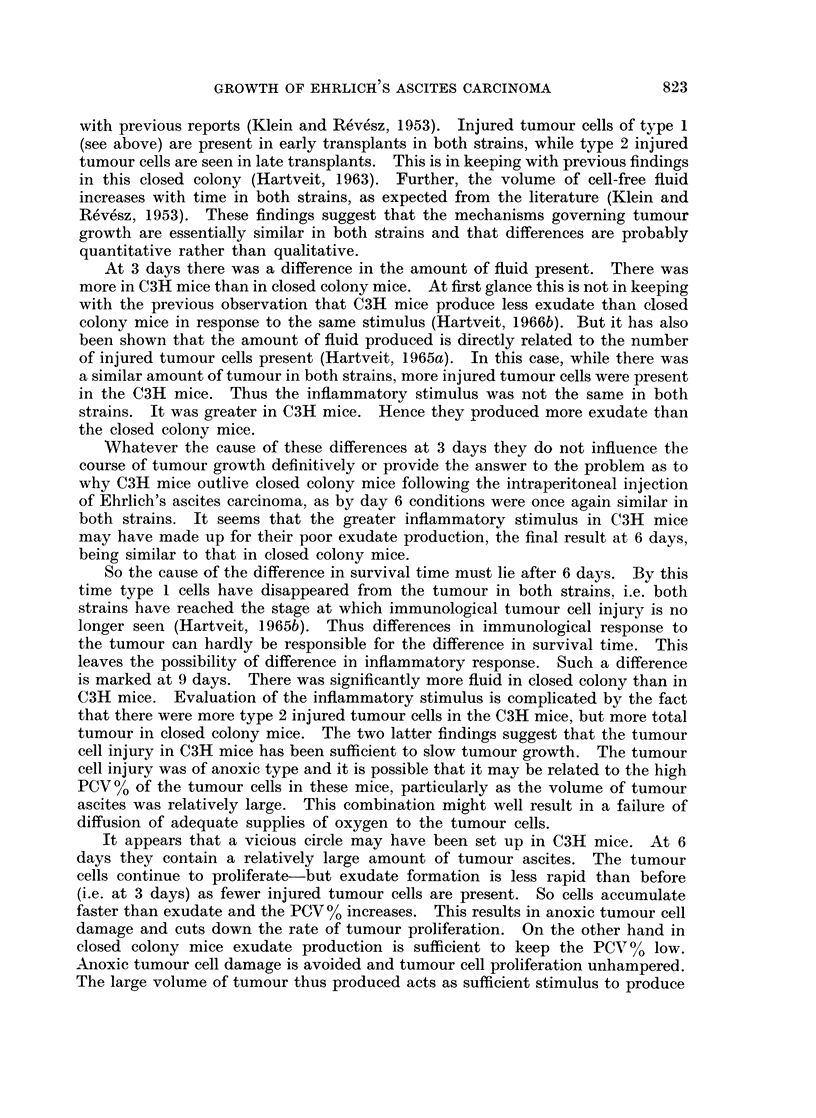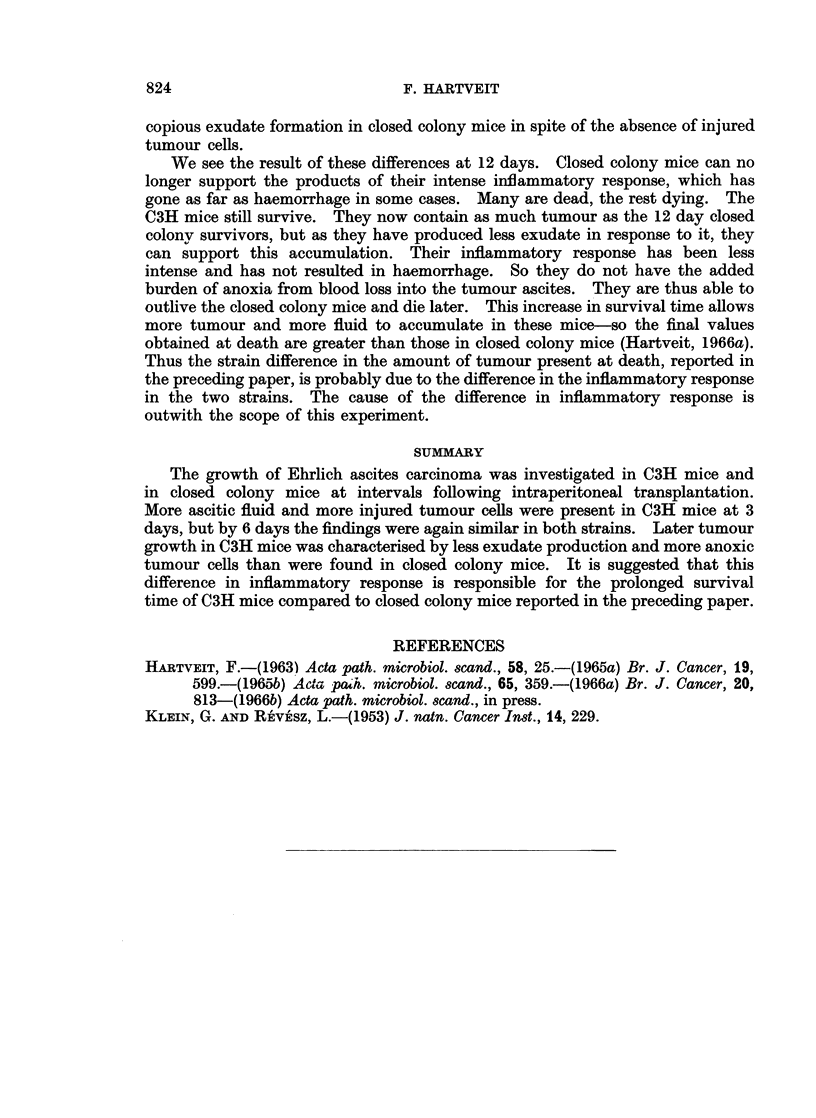# The growth of Ehrlich's ascites carcinoma in C3H mice and in mice of an unrelated closed colony. Variation in tumour growth, cytology and ascites formation.

**DOI:** 10.1038/bjc.1966.93

**Published:** 1966-12

**Authors:** F. Hartveit

## Abstract

**Images:**


### THE GROWTH OF EHRLICH'S ASCITES CARCINOMA IN C3H MICE AND IN MICE OF AN UNRELATED CLOSED COLONY

### VARIATION IN TUMOUR GROWTH, CYTOLOGY AND ASCITES FORMATION

F. HARTVEIT*

*From the Gade Institute, Department of Pathology, The University of Bergen, Norway*

Received for publication April 30, 1966

C3H MICE survive longer after the intraperitoneal injection of Ehrlich's ascites carcinoma than mice of our closed colony, and the final volumes of tumour and ascitic fluid formed differ (Hartveit, 1966*a*). The tumour cells cannot be held to be directly responsible for this difference as the same number of cells from the same transplant were given to all the mice. It is therefore probable that the difference lies in the host's response to the tumour cells.

It has recently been shown that C3H mice produce less peritoneal exudate than our closed colony mice following the intraperitoneal injection of Indian ink (Hartveit, 1966*b*). Theoretically the difference in survival time mentioned above may be a reflection of this difference in inflammatory response. The following study, in which the tumour ascites was investigated in groups of mice of both strains killed at 3 day intervals after tumour transplantation, was carried out to test this hypothesis.

### MATERIAL AND METHODS

The mice, C3H and mice of our closed colony, were of similar age and weight to those used in the previous experiment (Hartveit, 1966*a*). The tumour, the Ehrlich ascites carcinoma, was taken from a 10 day transplant in a female closed colony mouse as in the previous experiment.

### *Experimental procedure*

Four groups, each of 4 male and 4 female C3H mice, were set up. Corresponding groups of closed colony mice were also set up ; the first two groups as for C3H mice and two further groups with twice the number of mice. All the mice were given one intraperitoneal injection of 0·1 ml. whole tumour ascites (tumour cell dose $16 \times 10^6$).

The first two groups of C3H and closed colony mice were killed 3 and 6 days later, respectively. At 9 and 12 days some of the closed colony mice were dead

* Research Fellow, Norwegian Cancer Society.

### EXPLANATION OF PLATE

FIG. 1.—Three day transplant of Ehrlich's ascites carcinoma in C3H mouse. Leishman's stain. ×600. Note : Injured tumour cells, type 1 (←), lacking cytoplasm, with healthy tumour cells, two in mitosis.

FIG. 2.—Twelve day transplant of Ehrlich's ascites carcinoma in C3H mouse. Leishman's stain. ×600. Note : Injured tumour cells, type 2 (←), with degenerating cytoplasm and nuclei.

1 2

Hartveit.

but enough remained in these expanded groups to provide groups of 4 males and 4 females for comparison with the C3H mice at 9 days, while 4 males and 2 females were available at 12 days.

At death the tumour ascites was removed and measured. One ml. was centrifuged as described in the previous experiment and the total volume of the packed tumour cells, packed erythrocytes and the total cell-free fluid volume was calculated.

Films were made from the tumour ascites and stained with Leishman's stain. The percentage of injured tumour cells was calculated after counting approximately 200 tumour cells per slide. The morphology of the injured tumour cells was also studied as it has previously been shown that two types of morphological injury are seen in this tumour in closed colony mice (Hartveit, 1963). Type 1 cells (Fig. 1), showing what has been termed immunological type injury, occur early in the life history of the transplant. These cells show gross swelling of both cytoplasm and nucleus. The cytoplasm appears to be affected first and disintegrates before the nucleus, which may retain its basic structure while being stripped of cytoplasm. This stage is followed by a period in which very few injured tumour cells are seen, but later in the life history injured cells again appear. These type 2 cells (Fig. 2) can be differentiated from those described above, firstly by their morphology and secondly by the company they keep. Such damage has been attributed to anoxia. They too show swelling, but not to such a great extent as the type 1 cells. Cytoplasmic and nuclear degeneration appears to occur simultaneously, and the nuclear structure, plus nucleoli, may disappear before cytoplasmic changes are far advanced. These cells do not lose their cytoplasm as readily as type 1 cells. Secondly these cells are surrounded by cells showing lesser degrees of the same type of damage, while the cells round type 1 cells are healthy.

### RESULTS

The findings are given in Fig. 3 to 7. As none of the sex differences observed was statistically significant the values for both sexes have been pooled. The results are thus the means of 8 observations in all except the 12 day closed colony group in which only 6 observations were available.

The degree of statistical significance (t-test) of the differences between the 2 strains is shown at the foot of the figures, graded as follows:—

| | |
|---|---|
| — | $0{\cdot}1 > P > 0{\cdot}05$ and under |
| + | $0{\cdot}05 > P > 0{\cdot}02$ |
| ++ | $0{\cdot}02 > P > 0{\cdot}01$ |
| +++ | $0{\cdot}01 > P > 0{\cdot}001$ |
| ++++ | $0{\cdot}001 > P$ |

*Total packed tumour cells* (ml.), Fig. 3. The volume of tumour cells present increased with time in both strains up to day 9, but increased more quickly in closed colony mice. The difference between the strains was statistically significant at 9 days. Thereafter the amount of tumour present was lower in 12 day closed colony survivors, while the amount continued to increase in C3H mice.

*Injured tumour cells* (%), Fig. 4. The original tumour injected contained 2% injured tumour cells. At 3 days the number of injured cells had risen steeply in C3H mice, but not in closed colony mice. The strain difference was statistically significant at this time. At 6 days the number had remained about the same in the closed colony, and had dropped to a similar value in C3H mice. At 9 days there were again significantly more injured cells in C3H mice, while the closed colony value was little changed. At 12 days the number rose steeply in both

FIG. 3.—Total packed tumour cells (ml.) in C3H and closed colony mice related to time after the intraperitoneal injection of Ehrlich ascites carcinoma cells.

FIG. 4.—The percentage injured tumour cells (types 1 and 2, see text) in C3H and closed colony mice related to time after the intraperitoneal injection of Ehrlich ascites carcinoma cells.

strains, but more so in the closed colony which now contained significantly more injured tumour cells than the C3H mice.

Fig. 4 also indicates the morphology of the injured tumour cells. In both strains type 1 cells were seen at days 3 and 6, type 2 cells at days 9 and 12. These findings are further illustrated in the photographs (Fig. 1 and 2).

*Total packed erythrocytes* (ml.), Fig. 5. No blood was recorded in any of the C3H mice by this method. In closed colony mice no blood was recorded at 3

FIG. 5.—Total packed erythrocytes (ml.) in C3H and closed colony mice related to time after the intraperitoneal injection of Ehrlich ascites carcinoma cells.

days, but thereafter the volume of erythrocytes present increased with time, the strain difference being statistically significant at 12 days.

*Total cell-free fluid* (ml.), Fig. 6. At 3 days there was significantly more fluid in C3H than in closed colony mice. At 6 days the volume had increased in both strains and the strain difference was no longer significant. Thereafter the volume increased greatly in closed colony mice and more slowly in C3H mice, the strain difference being significant at 9 days.

*The packed cell volume (PCV) of the tumour cells* (%), Fig. 7. At 3 days the PCV was significantly higher in closed colony than in C3H mice. By day 6 it had risen in C3H to a level similar to that in the closed colony which had remained unchanged. At 9 days there was a drop in the PCV in the closed colony while that in the C3H mice continued to rise. Similar values were retained at 12 days. The strain difference was significant at 9 and 12 days.

### DISCUSSION

The present experiment shows that there are many differences in the progress of tumour growth with time following the intraperitoneal injection of Ehrlich's ascites carcinoma in C3H and closed colony mice, but at the same time there are also many similarities.

To consider the similarities first, the present experiment shows that the volume of tumour cells present increases with time in both strains. This is in keeping

FIG. 6.—Total cell-free fluid (ml.) in C3H and in closed colony mice related to time after intraperitoneal injection of Ehrlich ascites carcinoma cells.

FIG. 7.—Packed cell volume (PCV) of tumour cells, per cent, in C3H and closed colony mice related to time after the intraperitoneal injection of Ehrlich ascites carcinoma cells.

with previous reports (Klein and Révész, 1953). Injured tumour cells of type 1 (see above) are present in early transplants in both strains, while type 2 injured tumour cells are seen in late transplants. This is in keeping with previous findings in this closed colony (Hartveit, 1963). Further, the volume of cell-free fluid increases with time in both strains, as expected from the literature (Klein and Révész, 1953). These findings suggest that the mechanisms governing tumour growth are essentially similar in both strains and that differences are probably quantitative rather than qualitative.

At 3 days there was a difference in the amount of fluid present. There was more in C3H mice than in closed colony mice. At first glance this is not in keeping with the previous observation that C3H mice produce less exudate than closed colony mice in response to the same stimulus (Hartveit, 1966*b*). But it has also been shown that the amount of fluid produced is directly related to the number of injured tumour cells present (Hartveit, 1965*a*). In this case, while there was a similar amount of tumour in both strains, more injured tumour cells were present in the C3H mice. Thus the inflammatory stimulus was not the same in both strains. It was greater in C3H mice. Hence they produced more exudate than the closed colony mice.

Whatever the cause of these differences at 3 days they do not influence the course of tumour growth definitively or provide the answer to the problem as to why C3H mice outlive closed colony mice following the intraperitoneal injection of Ehrlich's ascites carcinoma, as by day 6 conditions were once again similar in both strains. It seems that the greater inflammatory stimulus in C3H mice may have made up for their poor exudate production, the final result at 6 days, being similar to that in closed colony mice.

So the cause of the difference in survival time must lie after 6 days. By this time type 1 cells have disappeared from the tumour in both strains, i.e. both strains have reached the stage at which immunological tumour cell injury is no longer seen (Hartveit, 1965*b*). Thus differences in immunological response to the tumour can hardly be responsible for the difference in survival time. This leaves the possibility of difference in inflammatory response. Such a difference is marked at 9 days. There was significantly more fluid in closed colony than in C3H mice. Evaluation of the inflammatory stimulus is complicated by the fact that there were more type 2 injured tumour cells in the C3H mice, but more total tumour in closed colony mice. The two latter findings suggest that the tumour cell injury in C3H mice has been sufficient to slow tumour growth. The tumour cell injury was of anoxic type and it is possible that it may be related to the high PCV% of the tumour cells in these mice, particularly as the volume of tumour ascites was relatively large. This combination might well result in a failure of diffusion of adequate supplies of oxygen to the tumour cells.

It appears that a vicious circle may have been set up in C3H mice. At 6 days they contain a relatively large amount of tumour ascites. The tumour cells continue to proliferate—but exudate formation is less rapid than before (i.e. at 3 days) as fewer injured tumour cells are present. So cells accumulate faster than exudate and the PCV% increases. This results in anoxic tumour cell damage and cuts down the rate of tumour proliferation. On the other hand in closed colony mice exudate production is sufficient to keep the PCV% low. Anoxic tumour cell damage is avoided and tumour cell proliferation unhampered. The large volume of tumour thus produced acts as sufficient stimulus to produce copious exudate formation in closed colony mice in spite of the absence of injured tumour cells.

We see the result of these differences at 12 days. Closed colony mice can no longer support the products of their intense inflammatory response, which has gone as far as haemorrhage in some cases. Many are dead, the rest dying. The C3H mice still survive. They now contain as much tumour as the 12 day closed colony survivors, but as they have produced less exudate in response to it, they can support this accumulation. Their inflammatory response has been less intense and has not resulted in haemorrhage. So they do not have the added burden of anoxia from blood loss into the tumour ascites. They are thus able to outlive the closed colony mice and die later. This increase in survival time allows more tumour and more fluid to accumulate in these mice—so the final values obtained at death are greater than those in closed colony mice (Hartveit, 1966*a*). Thus the strain difference in the amount of tumour present at death, reported in the preceding paper, is probably due to the difference in the inflammatory response in the two strains. The cause of the difference in inflammatory response is outwith the scope of this experiment.

### SUMMARY

The growth of Ehrlich ascites carcinoma was investigated in C3H mice and in closed colony mice at intervals following intraperitoneal transplantation. More ascitic fluid and more injured tumour cells were present in C3H mice at 3 days, but by 6 days the findings were again similar in both strains. Later tumour growth in C3H mice was characterised by less exudate production and more anoxic tumour cells than were found in closed colony mice. It is suggested that this difference in inflammatory response is responsible for the prolonged survival time of C3H mice compared to closed colony mice reported in the preceding paper.

### REFERENCES

HARTVEIT, F.—(1963) *Acta path. microbiol. scand.*, **58**, 25.—(1965*a*) *Br. J. Cancer*, **19**, 599.—(1965*b*) *Acta path. microbiol. scand.*, **65**, 359.—(1966*a*) *Br. J. Cancer*, **20**, 813—(1966*b*) *Acta path. microbiol. scand.*, in press.
KLEIN, G. AND RÉVÉSZ, L.—(1953) *J. natn. Cancer Inst.*, **14**, 229.